# The Use of AgNP-Containing Nanocomposites Based on Galactomannan and κ-Carrageenan for the Creation of Hydrogels with Antiradical Activity

**DOI:** 10.3390/gels10120800

**Published:** 2024-12-06

**Authors:** Marina Zvereva

**Affiliations:** A.E. Favorsky Irkutsk Institute of Chemistry, 664033 Irkutsk, Russia; mlesnichaya@mail.ru

**Keywords:** hydrogels, carrageenan, galactomannan, silver nanoparticles, antiradical activity

## Abstract

Series of composites containing 2.5–17.0% Ag and consisting of spherical silver nanoparticles with sizes ranging from 5.1 to 18.3 nm and from 6.4 to 21.8 nm for GM- and κ-CG-based composites, respectively, were prepared using the reducing and stabilizing ability of the natural polysaccharides galactomannan (GM) and κ-carrageenan (κ-CG). The antiradical activity of the obtained composites was evaluated using the decolorization of ABTS+^·^ solution. It was found that the IC_50_ value of a composite’s aqueous solution depends on the type of stabilizing ligand, the amount of inorganic phases, and the average size of AgNPs, and varies in the range of 0.015–0.08 mg·mL^−1^ and 0.03–0.59 mg·mL^−1^ for GM-AgNPs − κ-CG-AgNPs composites, respectively. GM-AgNPs − κ-CG-AgNPs hydrogels were successfully prepared and characterized on the basis of composites containing 2.5% Ag (demonstrating the most pronounced antiradical activity in terms of IC_50_ values per mole amount of Ag). It was found that the optimal ratio of composites that provided the best water-holding capacity and prolonged complete release of AgNPs from the hydrogel composition was 1:1. The influence of Ca^2+^ cations on the co-gel formation of the GM-AgNPs − κ-CG-AgNPs system, as well as the expression of their water-holding capacity and the rate of AgNPs release from the hydrogel carrier, was evaluated.

## 1. Introduction

Bacterial infections are still a significant cause (up to 30%) of annual deaths worldwide, including between 50 and 80% of deaths in young children [1,2,3]. In 2024, the WHO published an updated list of bacterial pathogens on its website, including 15 families of antibiotic-resistant bacteria identified in critical-, high-, and medium-priority groups, including bacteria of the genus *Salmonella* and *Shigella*, *Pseudomonas aeruginosa*, *Staphylococcus aureus*, *Neisseria gonorrhoeae* and *Enterococcus faecium*, group A and B streptococci, *Streptococcus pneumoniae*, and *Haemophilus influenzae*, which represent a high statistical contribution to the number of cases in low- and middle-income countries, particularly in vulnerable populations, including children and the elderly [4]. Due to the fact that antibiotics are the preferred method of therapy for bacterial infections in some cases, their widespread uncontrolled use has led to the appearance of multidrug-resistant bacterial strains and the emergence of superbacteria resistant to almost all antibiotics [5]. The use of nanoparticles in antimicrobial therapy provides promising opportunities to overcome challenges associated with traditional antibiotic treatment, including antibiotic resistance and limited drug penetration [6]. Interest in composites containing silver nanoparticles is because of the prospect of their use in biomedical applications due to the presence of their pronounced biological (antimicrobial, antifungal, antiviral, antiradical, hemostatic, etc.) activity, combined with their extremely low toxicity compared to ionic forms of silver, which were widely used earlier [7,8]. In this case, one of the main factors determining the low toxicity of this nanoparticle, compared to its ionic form Ag^+^, is the slow release of silver ions from the nanoparticle both due to the presence of a stabilizing shell on its surface and the need to overcome energy barriers to eliminate Ag^+^ ions from the Ag^0^ silver crystal lattice [9,10].

It is well known that the basis of the antimicrobial action of nanoparticles, and AgNPs in particular, is the parallel realization of several factors with the only possible result: their antimicrobial activity and cell damage. Thus, the main reasons for the antimicrobial action of nanoparticles are currently considered to be blocking the ionic transport of the cell, DNA damage, blocking protein–protein interactions, excess generation of reactive oxygen species (ROS), disruption of membrane integrity, release of ions from the surface of the nanoparticles, and destruction of biofilms [11,12]. The increased surface area of nanoparticles is a key factor contributing to their antimicrobial activity, primarily due to increased contact points between nanoparticle surfaces and bacterial cell membranes, as well as higher drug-loading capacity and enhanced adhesion to bacterial cells compared to similar bulk materials. These features promote the antimicrobial activity of nanoparticles, making them promising candidates for the development of effective antimicrobial therapeutic agents with targeted delivery to bacterial cells and the possibility of achieving therapeutic concentrations in the area of infection. At the same time, the balance maintained in normal physiological conditions between the constantly formed free radicals (prooxidants) and antioxidants neutralizing them is sharply disturbed under conditions of disease progress, including infectious diseases, in the direction of the predominance of prooxidants. This, in turn, is accompanied by disruption of natural metabolic processes and ultimately leads to the initiation of a number of biological programs determining cell death [13,14]. Maintaining an equilibrium balance between the components of this system is a mandatory condition for keeping the normal functioning of the cell, whereas in conditions of disturbance of this balance, practically the only way (along with elimination of the infectious agent) to restore the “pro-oxidant-antioxidant” equilibrium and neutralize excessively generated aggressive reactive oxygen species, as well as for preventive purposes, is to introduce additional amounts of antioxidants into the organism [15]. Thus, the active involvement of nanoparticles of a different nature in use in such areas as medicine, biology, ecology, and agriculture, mainly as antimicrobial agents, determines their potential use as antioxidant agents. To date, in vitro successfully demonstrated the expressed radical-binding ability of a number of nanoparticles of different chemical natures—Ag, Au, Pt, Pd, Cu, Fe_3_O_4_, ZnO, Se, NiO, etc.—against free the radicals DPPH^·^, ABTS^+·^, NO, O^2−^, etc. [16]. However, the presented studies are often limited to the detection of the radical-binding activity of nanoparticles without taking into account the sensitivity of the diagnostic value from the size characteristics of the nanoparticles or without considering the influence of ligands that stabilize their surface.

Currently, one of the promising methods for the therapy of wounds of various genesis, including burn wounds, is the practical use of hydrogel materials with drugs immobilized in the gel structure, including ones with antibacterial effects [17]. Due to the fact that the main properties of hydrogels for medical purposes are their inertness, biocompatibility, and ability to contain a significant amount of water, hydrogels have found wide application in medicine, including in the therapy of chronic and dehydrated wounds, surface burns, radiation damage of skin, abrasions, painful and superficial wounds, and granulating wounds, including those with scabs and necrotic tissues [18,19,20,21]. Taking into account the pronounced antibacterial activity of silver, its ions or nanosized silver are very often used as such an antimicrobial filler. In particular, silver-containing hydrogels with pronounced antimicrobial activity against *S. aureus*, *S. epidermidis*, *K. pneumoniae*, *E. coli*, *P. vulgaris*, *P. mirabilis*, and *C. albicans* were successfully obtained on the basis of the systems «Polyethylene glycol methyl ether methacrylate-dimethylamino ethyl methacrylate—N,N′-methylenebis(acrylamide)», «glycerol–carbopol-940—DMSO–triethanolamine», «polyacrylamide—N-methylenebisacrylamide», carboxymethyl cellulose, carboxymethyl chitosan, polyvinyl alcohol, polyacrylamide, polyethyleneimine, polyvinylpyrrolidone, and Pluronic F127 gel, as well as a variety of natural hydrogel matrices based on chitosan, sodium alginate, gelatin, glucomannan, guar gum, and others [22,23,24,25,26,27,28,29]. Due to their natural origin and intrinsic high water-holding capacity, natural polysaccharides have improved properties compared to synthetic polymers. Various hydrogels based on natural polymers have been successfully used in industry. However, hydrogels consisting of a single polymer cannot fully meet the requirements of industry in terms of both their properties and application characteristics. Therefore, in order to improve the properties of gels, new hydrogels based on two or more polymers have been actively developed in recent years. It should be noted in this regard that the synergetic system “κ-carrageenan-galactomannan” (κ-CG-GM), which does not lose its actuality, is used as a hydrogel base. The addition of GM to κ-CG solution depending on the GM/κ-CG ratio is accompanied by a significant modification of gel properties, in particular, an increase in its elasticity, strength, decrease in syneresis, increase in water-holding capacity, etc., observed in the presence of cations initiating the aggregation of κ-CG double helixes [30,31,32]. Numerous works devoted to the modification of κ-CG-based gels through the introduction of GM demonstrate the synergism and the prospect of developing these systems for the production of biocompatible materials, including for targeted drug delivery and therapy of wounds and burns [33,34,35,36,37,38,39]. In addition, the use of natural galactose-containing polysaccharides GM and κ-CG as a stabilizing shell for silver nanoparticles makes it possible to obtain water-soluble nontoxic nanomaterials with a pronounced complex of biological properties at high reproducibility. These biological properties of the obtained nanomaterials are determined both by polysaccharide (biocompatibility, membranotropicity, hypolipidemic activity, gel-forming properties) and the nanoparticles themselves (antimicrobial, antiviral, antitumor, antioxidant activity), including for combating both infectious agents and leveling the consequences of excessive generation of free radicals in the conditions of the natural immune response of the organism. Currently, there are already examples of the successful use of both GM and κ-CG for the synthesis of AgNPs. Thus, in [40,41,42,43,44], cases of null-valent silver nanoparticles with sizes from 5.7 to 100 nm, whose surfaces are stabilized by GM or CG macromolecules, are described. The obtained nanoparticles were characterized by acceptable stability in aqueous solutions (zeta potential over −30 mV) and demonstrated a set of pronounced antimicrobial, antitumor, metal-sensitive, etc., properties. Also, in an earlier work [45], a number of GM- and κ-CG-based silver-containing nanocomposites with antimicrobial properties were obtained. At the same time, these polysaccharides can serve simultaneously as a silver-reducing agent to the zero-valent state and as stabilizers of the formed nanoparticles. This makes it possible to obtain nanomaterials for biomedical research without the need to use highly toxic aggressive solvents and reducing agents. The evaluation of the possibility of creating hydrogel materials with anti-radical properties on the basis of GM- and κ-CG-stabilized AgNPs has not been studied before. This, together with the use of an ecological approach to the synthesis of hybrid organic–inorganic AgNP-containing nanomaterials with pronounced biological properties (previously discovered antimicrobial and, for the first time in these systems, antiradical) and the possibility of realizing the gel-forming potential of the “κ-CG-GM” system for the development of effective and safe hydrogel dressings determines the relevance and prospects of this work.

The aim of this work is the creation of AgNPs, hydrogels with antiradical activity based on the silver-containing nanocomposites GM-AgNPs and κ-CG-AgNPs, synthesized using the reducing and stabilizing ability of the natural polysaccharides galactomannan and carrageenan.

## 2. Results and Discussion

### 2.1. Synthesis of AgNPs Based on Galactomannan and κ-Carrageenan

The synthesis of GM-AgNPs and κ-CG-AgNPs composites was carried out in aqueous medium as a result of the reduction of silver ions to a zero-valent state only by matrices of GM and κ-CG natural polysaccharides. The reaction was initiated by increasing the pH of the reaction medium to 10–11 and the temperature to 60–90 °C. The duration of synthesis was varied from 15 to 50 min depending on the amount of silver introduced, the content of which in turn was varied by changing the Ag^+^/polysaccharide ratio (1 g) from 0.025 to 0.21. The possibility of realizing silver ion reduction by polysaccharide matrices is most likely provided either by direct oxidation of terminal aldehyde groups or primary alcohol groups present in the macromolecules of polysaccharides, or as a result of oxidation of reducing fragments with low molecular weight that detach from their macromolecules as a result of the process of “alkaline pilling” occurring in the presence of hydroxide anions introduced into the reaction medium [46]. The latter assumption is supported by the previously observed significant change in molecular weight (Mw, degree of polydispersity) characteristics of the obtained Ag^0^-containing nanocomposites compared to the original polysaccharides [47].

As a possible mechanism of silver nanoparticle formation in polysaccharide matrices, we can assume the sequential condensation of reduced silver atomic particles reaching a critical concentration in the reaction medium with the formation of a nuclei of a new solid phase. Subsequent formation of nanoparticles is probably realized due to the continuous growth of formed nuclei as a result of the sorption of silver atoms on their surface, as well as due to the redissolution of unstable nuclei. Completion of the process is probably caused by the adsorption of polysaccharide macromolecules GM or κ-CG on the surface of the growing nanoparticle, limiting the diffusion of atomic silver to the surface of the nanoparticle, as well as a significant decrease in the concentration of atomic silver in the reaction medium due to the completion of ion reduction processes. It was found that on the basis of GM and κ-CG, it was possible to obtain nanocomposites with silver content up to 17.0% and 12.0%, respectively. Presumably, the observed differences in the maximum possible silver content in nanocomposites obtained on the basis of GM and κ-CG may be due to their molecular mass and structural characteristics, which in turn determine such important parameters that determine the dynamics of synthesis as the viscosity of the reaction medium, the flexibility of macromolecules, and their conformation in the reaction medium [47,48]. The high viscosity of aqueous solutions of GM and κ-CG, as well as the pronounced polyelectrolyte nature of κ-CG and the dependence of the conformation of its macromolecules on the addition of electrolytes, also have an impact on the result of the synthesis of nanocomposites [49]. This, in turn, contributes to the existence of diffusion limitations in the reaction medium that limit the rate of arrival of reduced Ag^0^ atoms to the surface of growing particles, as well as the sorption of polysaccharide macromolecules on their surface at the stabilization stage. The consequence of this is probably a discrepancy between the growth rate of nanoparticles and the rate of their stabilization and loss of stability of the formed nanoparticles. The presence of a sufficiently large amount of hydroxide ions and Ag^+^ and Na^+^ ions in the reaction medium under conditions of obtaining composites with high (more than 12%) silver content in the composition is likely to be accompanied by an ion-induced conformational transition ball double helix of κ-CG macromolecules, i.e., ordering the structure and the impossibility of their further participation in the stochastic stabilization of the surface of silver nanoparticles [50]

### 2.2. Structure and Nanomorphology Characterization of GM- and κ-CG-Based AgNPs

#### 2.2.1. Powder X-Ray Diffraction Analysis of GM-AgNPs and κ-CG-AgNPs Composites

**Figure 1:**
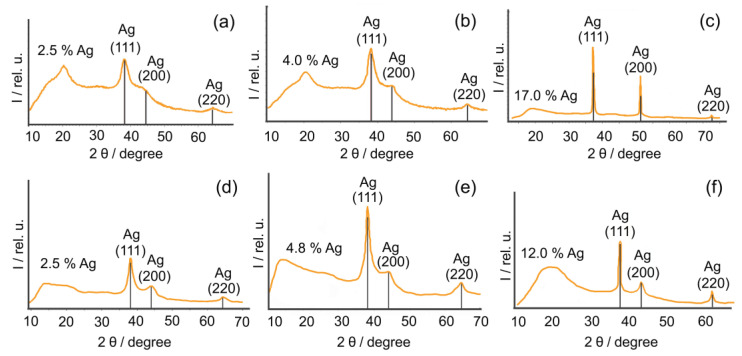
XRD diffractograms of AgNP composites based on GM (**a**–**c**) and κ-CG (**d**–**f**) with different percentages of silver nanoparticles.

The phase structure of nanocomposites was identified on the basis of diffractogram data, which clearly differentiate the halo of the amorphous phase of polysaccharide matrices in the region of 10–30° 2θ angles and broadened reflexes in the region of 38.1°, 44.2°, and 64.3° 2θ corresponding to the (111), (200), and (220) planes of the face-centered cubic crystal lattice Ag^0^ (a = b = c = 4.086 Å with space group Fm-3m (225) (JCPDS 99-0094) (Figure 1).

According to the obtained X-ray data, an increase in the quantitative silver content in the nanocomposites is accompanied by a narrowing of the reflexes and an increase in the average value of the coherent scattering region in AgNPs. In particular, for all of the obtained samples of nanocomposites, the average sizes of crystallites formed in polysaccharide matrices were calculated using the Scherrer formula, the value of which varies depending on the type of matrix and the quantitative content of silver in the composition of the composite [51]. The main characteristics of the crystalline phase of silver-containing nanocomposites are presented in more detail in Table 1.

The largest silver particle sizes of 6.4–24.8 nm for composites containing 2.5–12.0% Ag were recorded in the case of using sulfated polysaccharide κ-CG as a reducing and stabilizing matrix. GM-based composites containing 5.1–18.3 nm nanoparticles as the inorganic phase for composites containing 2.5–17.0% Ag were obtained. Probably due to the presence of anhydrocycles and sulfogroup in position 4 of β-D-galactopyranose units, some amounts of hydroxyl groups in the macromolecules of κ-CG were not available for participation in the processes of silver reduction or stabilization of the surface of the formed nanoparticles. This may determine the smaller amount of silver able to be reduced in the polysaccharide matrix of κ-CG, and the larger size of the formed nanoparticles compared to GM.

In addition, based on the assumption of the spherical shape of the particles, as well as their average sizes obtained using XRD analysis, the values of the specific surface area (S*_sp_*. cm^2^/cm^3^) of silver nanoparticles in all of the obtained nanocomposites were calculated. It was found that an increase in the average size of the nanoparticles was accompanied by a decrease in the value of S*_sp_*, and the most pronounced decrease (by an order of magnitude) was observed in cases where the size of the nanoparticles exceeded 6.4 nm (Table 1). As a result, the increase in silver atoms forming the whole volume of nanoparticles formed in polysaccharide matrixes of GM and κ-CG with increases in their average size was accompanied decreases in the proportion of atoms located on the surface of the nanoparticles from 30% for particles having the size of 5.1 nm to 7% in the case of the largest (24.8 nm) nanoparticles formed in the polysaccharide matrix of κ-CG. The modern concept of the phenomenon of the “nanoscale state”, including high percentages of surface atoms in nanoparticles, determines the occurrence of their unique physical and chemical properties.

Thus, surface atoms make a major contribution to the thermodynamic characteristics of solids and, for nanoparticles, largely determine their structural transitions and melting points [52]. In addition, the size of some nanoparticles is comparable to the size of molecules, which in turn determines the specificity of the kinetics of chemical processes on their surface [53]. The increase in S*_sp_* values when particle size decreases is accompanied by the appearance of numerous defects and intercluster stresses in nanostructures; therefore, the primary task is the directed regulation of the nanomaterials’ dimensionality in the process of their synthesis, including by means of the matrix limitation of nanoparticle growth in the process of their stabilization [54].

#### 2.2.2. TEM Study of GM-AgNP and κ-CG-AgNP Composites’ Nanomorphology

**Figure 2:**
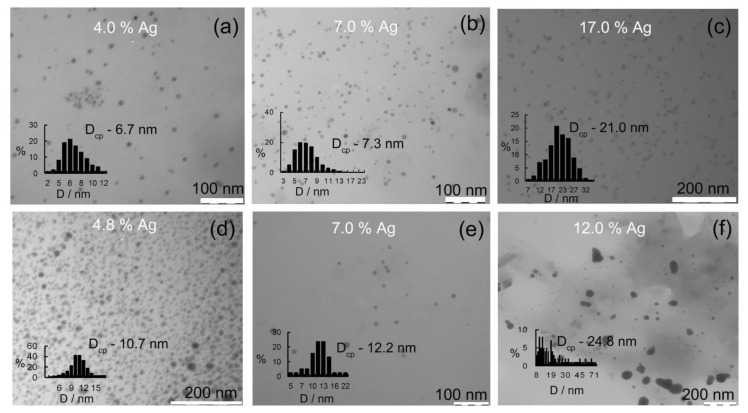
Microphotographs (TEM) of AgNP composites based on GM (**a**–**c**) and κ-CG (**d**–**f**) with different percentages of silver.

It was found that nanocomposites are formed in the form of particles distributed in polysaccharide matrices with sizes of 3–71 nm, the shape of which are similar to spheres (Figure 2). The analysis of the obtained data allowed us to determine that, in composites GM-AgNPs containing 4.0% of silver, there were identified particles whose shape was almost spherical with sizes of 2–12 nm and an average diameter value of 6.7 nm. At the same time, only 19% of all particles had a size close to the average value. Increasing the amount of silver in the composites up to 7.0% was accompanied by an increase in the average diameter of silver nanoparticles up to 7.3 nm, as well as by a significant widening of the size range of nanoparticles up to 3–21 nm. In the case of Ag-GM composites containing the maximum possible (17.0%) amount of silver in the composition, the average diameter value was already 21.0 nm, while the size range of particles identified by us expanded to 7–32 nm. The largest size of silver nanoparticles was found in the case of κ-CG. Thus, the introduction of 4.8% silver into the composition of κ-CG was accompanied by the formation of particles whose shape was almost spherical, and the sizes varied in the range of 4.0–21.0 nm, with an average value of 10.7 nm. Increasing the quantitative content of silver in the composite up to 7.0% was characterized by an increase in the average size of silver nanoparticles up to 12.2 nm and by the broadening of polydispersity in the range of 5.0–22.0 nm. If the nanocomposite contained the highest amount of silver capable of stabilization by κ-CG (12.0%), the formed nanoparticles were characterized by both the deviation from the spherical shape and a very wide range of sizes from 8.0 to 78.0 nm.

That is, under conditions including a high content of silver nitrate in respect to the amount of κ-CG in the reaction medium, in addition to the main stages of nucleation and growth of nanoparticles occurring probably by the Lamer mechanism, a number of mechanisms leading to the coalescence of nanoparticles (aggregation) were realized. This is also evidenced by the presence of a fraction of small particles and a fraction of large particles, the size of which varied in the range of 45–78 nm on the micrographs of this sample. The observed coalescence of several nanoparticles into an aggregate in the presence of a stabilizer in the reaction medium was possible in case of its insufficient quantity. This, in turn, causes contact with the stabilizer-free surface of several nanoparticles and the completion of the silver lattice, in association with the particles, into a single polycrystalline or oriented aggregate deviating from the spherical shape. The reason for the unexpressed stabilizing ability of κ-CG in conditions including an excess precursor in the inorganic phase may also be in its polyelectrolyte nature. Firstly, a large amount of silver nitrate introduced into the reaction medium for the synthesis of composites containing more than 10% Ag requires more hydroxide ions to shift the pH to the alkaline region and complete conversion of Ag^+^ to Ag^0^ in the process of silver reduction by κ-CG macromolecules. The increase in the ionic strength of the solution contributes to a change in the conformation of macromolecules of κ-CG itself due to the deprotonation of its hydroxyl groups under conditions of excess OH- ions, and the transition of the conformation of κ-CG from unfolded macromolecules or loose balls to densely packed globules that are probably not able to fully interact with the surface of the formed nanoparticles and provide their steric stabilization. In addition, the smaller number of hydroxyl groups in κ-CG macromolecules also does not promote the stabilization of the nanoparticle’s surface by coordination interactions of the silver nanoparticle’s surface with κ-CG hydroxyl groups.

The analysis of the shape of dispersion distribution of silver nanoparticles in polysaccharide matrices suggests the influence of synthesis conditions, namely the Ag^+^/polysaccharide ratio, as well as the type of polysaccharide on the character of AgNPs size distribution as a result of the realization of fundamentally different processes occurring at the stage of growth and maturation of nanoparticles. The size distribution in the polysaccharide matrix of GM under conditions of nanocomposites containing 2.5–7.0% Ag is close to log-normal and is characterized by positive asymmetry with the asymmetry factor (*As*) varying in the range of 1.25–1.75. According to the currently existing ideas about the mechanisms of growth and maturation of nanoparticles in condensed media, the distribution of nanoparticle dispersity close to the normal type indicates that nanoparticles grow as a result of the sequential condensation of atomic silver to the surface of the growing particle and not by stochastic aggregation of the formed nuclei of a new phase or silver clusters [55,56]. Spherical particles with minimal surface area per unit mass (volume) are formed in the absence of factors that promote or inhibit the growth of particles along certain faces of their crystal lattice. The form of disperse distribution is characterized by negative asymmetry with an *As* value of 0.9 in cases where the quantitative silver content in the GM-Ag composites increases up to 17.0%.

It should be noted that for composites based on κ-CG, the negative asymmetry of the disperse distribution of nanoparticles is characteristic already at 7.0% of silver in the composition. The start of the process showing the prevalence of the fraction with larger particle size under conditions of increased Ag^+^/polysaccharide ratios in nanocomposite synthesis, as well as a larger number of formed Ag^0^ nanoparticles in the volume of the reaction medium, is probably due to the Ostwald ripening process, with the dissolution of the fraction of smaller particles (due to their increased solubility) and the completion of growing nanoparticles of larger diameters due to the released Ag^0^ atoms.

The preservation of the almost spherical shape of silver nanoparticles, even in the case of GM-Ag nanocomposites containing 17.0% silver proves, the realization of this mechanism. In the case of κ-CG-Ag nanocomposites (12.0% Ag), the shapes of nanoparticles forming the second (large-size) fraction deviate from spherical in contrast to the first (low-size) fraction. This, together with the bimodal character of the disperse distribution of nanoparticles, most likely indicates the realization of growth and maturation of AgNPs due to the coalescence of some fraction of nanoparticles in the reaction medium with the formation of aggregates visualized on the TEM images. So, the particle growth under the conditions of using GM and κ-CG as a nanostabilizing matrix and low saturation of the reaction medium with precursor ions is probably due to the sequential adsorption of reduced Ag^0^ atoms on the surface of the nanoparticles. Taking into account the fact that the silver reducing agent is the polysaccharide itself, the reduction process is somewhat slower than when using standard strong reducing agents (NaBH_4_, N_2_H_4_·H_2_O, etc.). Therefore, the formation of monomeric units forming nanoparticles is also distributed in time. Theoretically, Ag^0^ atoms formed after the nucleation stage due to the impossibility of reaching a critical concentration of phase formation and the presence of a new phase, growing nanoparticles in the reaction medium (i.e., heterogeneity of the process), are forced to sorb on their surface, i.e., to participate not in the additional formation of new crystallization centers, but in increasing the size of nanoparticles already present in the reaction medium. This can probably explain the increase in the particle size in the reaction medium when the amount of inorganic nanophases in the composite is increased [57]. A more complex behavior between monomers and particles is observed under conditions which include the supersaturation of the reaction medium with precursor ions with increasing Ag^+^/polysaccharide ratios. That is, the growth of particles is explained not only by the addition of monomers (reduced silver atoms) to the solution, but also by the interaction and coalescence of particles with each other, with the subsequent coagulation of cluster fragments and formation of aggregates.

#### 2.2.3. Optical Properties of GM-AgNPs and κ-CG-AgNPs Composites

**Figure 3:**
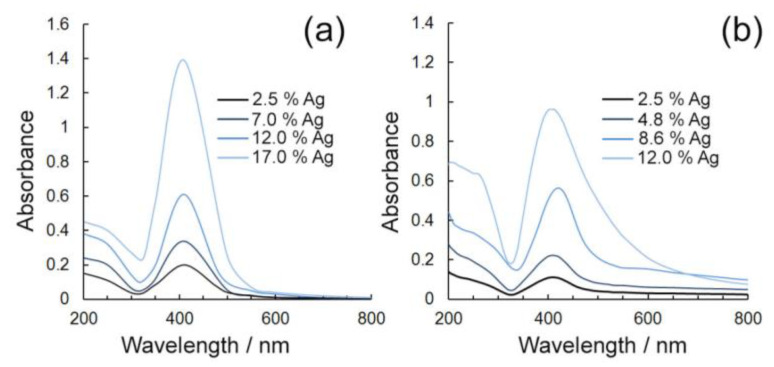
Absorption spectra of 0.05% aqueous solutions of AgNP composites based on GM (**a**) and κ-CG (**b**) with different silver contents.

The spectra of aqueous solutions obtained using AgNP composites has an intense absorption maximum at a wavelength of 404–410 nm (Figure 3) due to the collective excitation of silver conduction electrons in the light wave field—surface plasmon resonance (SPR). The presence of this effect confirms not only the zero valence of silver in the nanoparticles, but also their close to spherical geometry due to the presence of only one maximum plasmon absorption of aqueous solutions of nanocomposites in the visible region of the spectrum [58].

**Figure 4:**
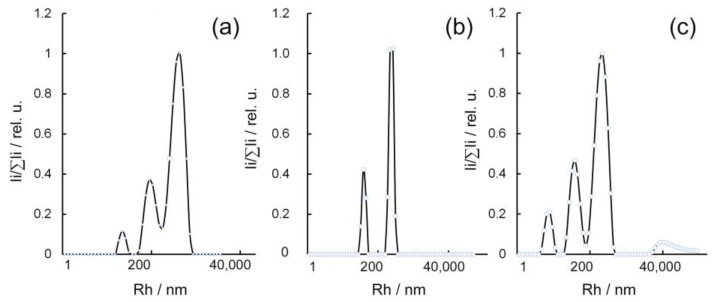
Intensity distribution of Rh particles in 0.1% aqueous solutions of κ-CG (**a**) and κ-CG-AgNPs composites with 2.5% and 7.0% Ag (**b**,**c**), respectively.

The dependence of the intensity of the plasmon absorption peak on the amount of silver introduced into the nanocomposite has been established experimentally. In particular, it was demonstrated that the increase in the quantitative content of silver in the nanocomposite is accompanied by an increase in the intensity of plasmonic absorption, which also indicates, in combination with the data of “classical” methods of characterization of nanocomposite structure (TEM, XRD), the possibility of directional variation in characteristics (particle size, optical properties) by changing the conditions of synthesis (in this case, the ratio of precursors) due to their direct dependence on the percentage of silver in the composition. According to the data presented in Figure 4, an increase in silver content in the nanocomposite is accompanied by an increase in the intensity of plasmonic absorption due to an increase in the number of nanoparticles containing generalized electrons interacting with a certain range of electromagnetic radiation.

#### 2.2.4. Characterization of GM- and κ-CG-Stabilized AgNPs Using the Dynamic Light Scattering Method

According to DLS data, aqueous solutions of κ-CG in the distribution of Rh values in the particle-scattering intensity are characterized by the presence of three fractions (Figure 4). The fast particle fraction with an average Rh value of 38.9 nm agrees well with the previously presented data on Rh values for κ-CG [59,60]. This Rh value most likely corresponds to the loose ball conformation (statistical ball) as the most characteristic conformation for a macromolecule in the absence of interchain interactions. The statistical ball conformation has maximum entropy compared to the rod or globule conformation, so it appears spontaneously. Externally, it looks like the spontaneous “folding” of a rod or “unfolding” of a globule. In addition to the fast mode, the distribution also contains two other slow modes with average Rh values of 195 nm and 1201 nm, most likely corresponding to the intermolecular aggregates of κ-CG macromolecules, usually observed in highly charged polyelectrolytes, in which attractive interactions between polyions are mediated by condensed counterions [61]. The introduction of silver nanoparticles into κ-CG during the synthesis of composites is accompanied by a change in the Rh distribution of particles present in the aqueous solution of the κ-CG-Ag nanocomposite (2.5% Ag), namely, the disappearance of a mode with an average Rh value of about 38.9 nm and the presence of two new modes with average Rh values of 73.7 nm and 678.0 nm. Probably, the first fraction of particles corresponds to silver nanoparticles, the surfaces of which are covered by hydrated κ-CG macromolecules interacting with each other by electrostatic attraction.

The second fraction of particles with an average Rh value of about 678.0 nm most probably corresponds to aggregates and macromolecules of κ-CG and aggregates of Ag particles covered by a κ-CG stabilizing shell. Increasing the quantitative content of silver in the composite up to 4.8% is accompanied by the preservation of a bimodal distribution and average Rh values of particles present in its solution. Further increases in silver content in composites up to 7.0% are accompanied by the transfer of Rh size distribution of particles from the bimodal to multimodal type and the separation of three modes. The first fast mode, with an average Rh value of about 12.0 nm, has not been previously identified either in the original κ-СG or in composites with less silver. The appearance of this particle fraction in the aqueous solution of the κ-CG-Ag composite (7.0% Ag) is most probably caused by the partial depolymerization of κ-СG macromolecules in the process of nanocomposite synthesis, namely, in the process of Ag^+^ ions’ reduction to the zero-valent state, including by detachment of reducing terminal moieties. The additionally depolymerized κ-CG molecules obtained are also able to participate in the surface stabilization of Ag^0^ nanoparticles. The second mode, identified in the Rh distribution of the particle-scattering intensity, has an average Rh value of 73.6 nm and probably, as in previous cases, corresponds to aggregates of κ-CG macromolecules with Ag nanoparticles in their structure. The third fraction of particles, with an average Rh value of about 553.0 nm, most probably corresponds to aggregates of κ-CG macromolecules and aggregates of Ag particles covered with a stabilizing shell of κ-CG. The increase in the silver content in the composite up to 8.6% is accompanied by a fast mode in the Rh distribution of particles according to their scattering intensity corresponding to the particle fraction with an average Rh value of 2.9 nm. In addition, the particles in this fraction may be depolymerized fragments of κ-CG formed during the synthesis of nanocomposites. Such fragments probably do not participate in the stabilization of the nanoparticle’s surface and are present in the nanocomposite solution in a free ball-like state. In addition to such low-dimensional components, a fraction of particles with an average Rh value of 14.5 nm was identified in the solution, also probably corresponding to partially depolymerized κ-CG macromolecules formed during the synthesis of nanocomposites and participating in the stabilization of nanoparticles, whereas the fractions with average Rh values of 73.4 and 451.0 nm probably correspond to aggregates of κ-CG macromolecules, including in combination with aggregates of κ-CG-stabilized Ag nanoparticles.

Due to polydispersity, the presence of many particle fractions with different Rh values, and the difficulty of qualitative interpretation, the determination of Rh for GM and its composites has not been carried out. Therefore, it was decided to only characterize κ-CG-based nanocomposites using the DLS method.

### 2.3. Investigation of GM-AgNPs and κ-CG-AgNPs Composites’ Antiradical Activity

**Figure 5:**
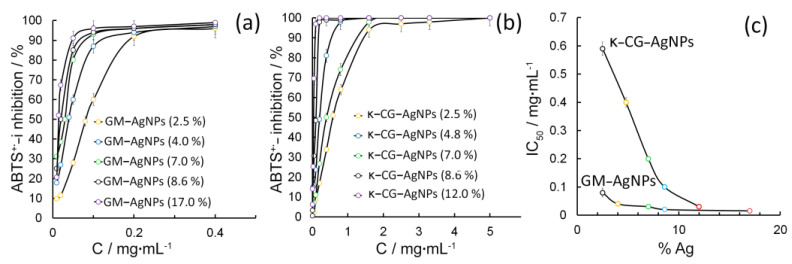
Radical-binding ability of GM-AgNPs (**a**) and κ-CG-AgNPs (**b**) composites against ABTS^+^·; dependence of the IC_50_ parameter on the silver concentration in the GM and κ-CG-based nanocomposites (**c**). Error bars are hidden in the bar when not visible; data are mean ± SD, *n* ≥ 3.

The study of antiradical activity of the obtained nanocomposites was carried out with the use of a standard test system based on the cation-radical ABTS^+·^. Aqueous solutions of ascorbic acid and pure polysaccharides of GM and κ-CG were used as controls. It was found that the value of inhibition of ABTS^+^· by pure polysaccharide solutions in the concentration range of 0.01–5 mg·mL^−1^ did not exceed 6%. This indicates the low antiradical activity of GM and κ-CG polysaccharides. The addition of 2.5–17.0% of AgNPs into their composition is accompanied by the appearance of a pronounced (up to 100%) radical-inhibiting ability of the obtained nanocomposites (Figure 5a). At the same time, the value of the concentration providing the inhibition of 50% of radicals in the volume of the analyzed sample (IC_50_) depends on the percentage of silver in the composites and on the type of stabilizing matrix. In particular, an increase in the quantitative content of AgNPs in the GM-AgNPs composite from 2.5 to 17.0% is accompanied by a decrease in the IC_50_ value from 0.08 to 0.015 mg·mL^−1^ (Figure 5c).

An increase in the amount of silver in κ-CG-AgNPs composites from 2.5 to 12.0% is accompanied by a decrease in IC_50_ from 0.59 to 0.03 mg·mL^−1^ (Figure 5b). The analysis of the obtained data allows us to draw conclusions about the more pronounced antiradical activity of GM-based nanocomposites in comparison with κ-CG-based composites, as well as the key role of AgNPs in the demonstration of antiradical activity by nanocomposites. Taking into account the low (2.5–17.0%) content of silver in the composites, as well as the exclusive dependence of anti-radical activity of composites only on silver nanoparticles, the obtained values, in terms of per mole of active substance (i.e., silver), are comparable with the IC_50_ of ascorbic acid. It was found that, for 50% neutralization of 3 × 10^−7^ mol of ABTS^+·^, in the analyzed sample, a volume of 5.5 × 10^−8^–7.0 × 10^−8^ mol of silver in GM-AgNPs composites or 4.1 × 10^−7^–1.0 × 10^−7^ mol of silver in κ-CG-AgNPs composites are needed.

The analogous value for ascorbic acid is 9 × 10^−7^ mol. The more pronounced antiradical activity of GM-AgNPs composites is probably due not only to the smaller size of the AgNPs formed in the GM matrix, but also to the contribution of κ-CG sulfogroups to the passivation of the AgNPs surface.

### 2.4. Study of Water-Holding Capacity of AgNPs-Containing Hydrogels Based on GM-CG System

Earlier, in [47], a decrease in the molecular weight of GM and κ-CG during the synthesis of silver nanocomposites was demonstrated. Moreover, this decrease depended on the amount of metal introduced into the composite, reaching 65–70% in the case of composites with a large amount of inorganic nanophase (12–17%). The decrease in molecular weight is probably due to the depolymerization processes catalyzed by hydroxide ions present in the reaction medium in concentrations directly correlated with the concentration of AgNO_3_ precursors. The electron acceptor carbonyl and carboxyl groups appearing as a result of redox interactions in the macromolecules of polysaccharides, due to the negative inductive effect, reduce the electron density on the oxygen atom of the glycosidic bond, which, in turn, causes its higher susceptibility to OH^−^ ions and accelerate the process of alkaline depolymerization of macromolecules, accompanied by a decrease in the molecular weight of these objects. The decrease in molecular weight also leads to changes in such important parameters as gelation time and gelation concentration (for κ-CG-based composites), increasing their values. Thus, the introduction of 2.5–12% AgNPs into the carrageenan matrix is accompanied by a decrease in the value of the average viscous molecular weight of the composites to 900–320 kDa and, respectively, an increase in the gelation concentration from 1.5 to 2% and gelation time from 11 to 20 min. In addition, the above-presented TEM and XRD data indicate an increase in the AgNPs average size when the amount of inorganic phases in the composite increases. Due to the direct dependence of the physicochemical activity of silver nanoparticles on their size, and the gel-forming ability of κ-CG on its molecular weight, it was decided to use composites with 2.5% AgNP content to create hydrogels.

The biomedical use of hydrogels, especially as wound dressings, is based on their ability to maintain a moist environment on the wound surface, thus promoting its healing. High water content (up to 99%) determines excellent biocompatibility and flexibility of hydrogels, making their use as comfortable and efficient. In other words, water-holding capacity is one of the key parameters characterizing the potential of hydrogels in biomedicine. The estimation of hydrogel water-holding capacity is important for evaluating the potential of hydrogels and selecting their composition in accordance with the task [62]. In this work, a series of AgNPs-containing hydrogels based on the GM-κ-CG system with a variable component ratio (GM-AgNPs/κ-CG-AgNPs) from 0 (only κ-CG-AgNPs) to 4 were prepared by mixing 1.5% solutions of previously obtained and characterized AgNP-containing nanocomposites based on these polysaccharide matrices. The study of their water-holding capacity was carried out at a temperature of 30 °C as an average value between the temperature of the human body on its surface and room temperature, i.e., in the temperature range of the possible exploitation of hydrogels.

**Figure 6:**
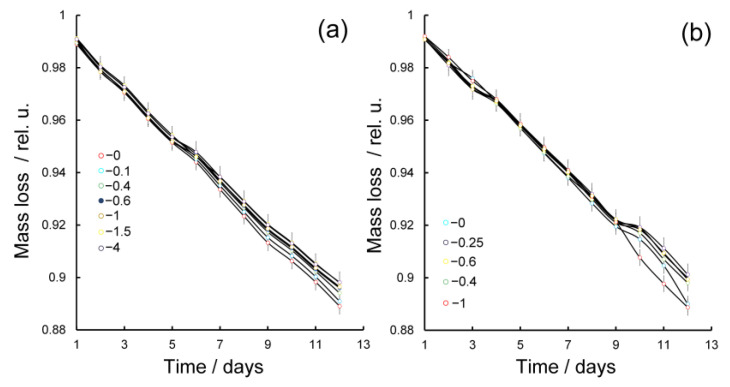
Water-holding capacity of AgNP-containing hydrogels based on the GM-κ-CG system in the absence (**a**) and presence (**b**) of Ca^2+^ ions. Error bars are hidden in the bar when not visible; data are mean ± SD, *n* ≥ 3.

The results are presented in Figure 6a. It was found that at this temperature, regardless of the ratio of GM-AgNPs/κ-CG-AgNPs, all obtained hydrogels lost insignificant amount of water until the 12th day of the study. The highest (11.1%) moisture loss was registered in the case of hydrogel based on κ-CG-AgNPs only, whereas the addition of the GM-AgNPs composite to it was accompanied by reduced values in moisture. The lowest value of moisture loss (10.1%) was observed in the case of the GM-AgNPs/κ-CG-AgNPs system with the ratio of components being 1:1. The introduction of GM-based composites into the κ-CG gel composition at equal quantitative ratios was accompanied not only by the possibility of increasing the antiradical activity of these hydrogel systems due to the more pronounced radical-binding ability of GM-AgNPs composites in comparison with κ-CG-AgNPs, but also the possibility of increasing their water-holding capacity.

The addition of K^+^ and Ca^2+^ cations is often used to modify the properties of hydrogels, including those based on κ-CGs. Their introduction stimulates rapid and strong structuring of gels with the formation of a spatial three-dimensional network. The resulting gels are usually characterized by higher values in the elastic modulus, high optical transparency, and fine network structures [63]. Many works have been devoted to the study of the influence of K^+^ and Ca^2+^ cation additives on the viscoelastic and mechanical properties of hydrogel systems based on GM-κ-CG. In this study, it was of interest to investigate Ca cation addition not only on the strength and elasticity of gels, but also on their water-holding capacity. According to the data obtained, the introduction of Ca^2+^ cations into GM-AgNPs/κ-CG-AgNPs hydrogel systems with different component ratios was also accompanied by an insignificant amount of moisture loss. The highest moisture loss (11.1%) was unexpectedly recorded in the case of hydrogels with a GM-AgNPs/κ-CG-AgNPs 1:1 ratio, whereas the lowest moisture loss (9.9%) was recorded for the system with the ratio of GM-AgNPs/κ-CG-AgNPs being 0.25; that is, the co-structuring of the gel in the GM-κ-CG system and in the presence of Ca^2+^ cations is characterized by a strong binding of water either inside the spatial network of the κ-CG macromolecules with inclusions of GM macromolecules or inside the interpenetrating networks of the aggregated double helices of κ-CG and GM macromolecules. Taking into account the presence of a more pronounced water-holding capacity of the GM-AgNPs/κ-CG-AgNPs system in the case of a small amount of GM (GM/κ-CG 0.25), it is more favorable to consider, as a possible mechanism of κ-CG gel formation in the presence of GM and Ca^2+^ cations, the “closing” inside the spatial network of the structured gel of the κ-CG inclusions to GM aqueous solution. The water-holding capacity of hydrogels based on GM-AgNPs/κ-CG-AgNPs could not be significantly modified by Ca^2+^ additives.

### 2.5. Study of the Effect of GM-AgNPs/κ-CG-AgNPs Ratios on the Elastic Modulus of Ca^2+^-Enhanced Hydrogels

**Figure 7:**
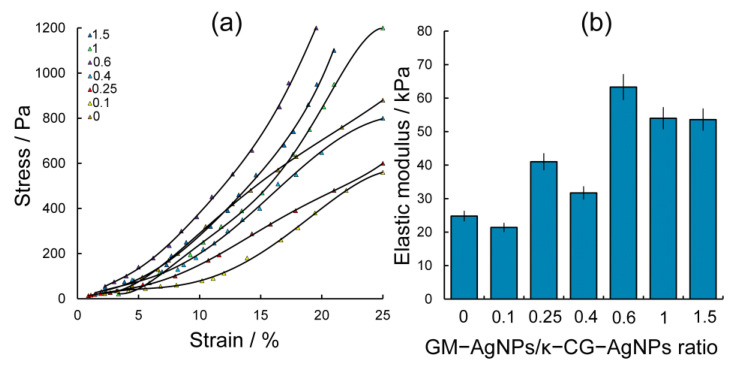
Effect of GM-AgNPs-κ-CG-AgNPs ratio on the stress–strain dependence of hydrogels (**a**) and their elastic modulus (**b**).

The most satisfactory results in terms of water-holding capacity were obtained for hydrogels based on the GM-AgNPs-κ-CG-AgNPs system. However, the obtained hydrogels in the absence of Ca^2+^ ion addition were characterized by brittleness and rather long gelation times (more than 20 min). The addition of Ca^2+^ cations not only accelerated gelation by the 13th minute, but also provided strong gels, which were further investigated using the standard method of uniaxial compression to determine the effect of the ratio of components on such important characteristics as the elastic modulus of the gel and the strain–stress dependence of its deformation. The results are presented in Figure 7.

Thus, the highest values of the hydrogel elastic modulus were found for samples with GM-AgNPs/κ-CG-AgNPs ratios of 0.6, 1, and 1.5. This indicates a significant (2–2.5 times) increase in the elasticity of hydrogels obtained by combining GM-AgNPs/ κ-CG-AgNPs composites in comparison with hydrogel samples obtained using only κ-CG-AgNPs. It is known that κ-carrageenan is able to form brittle gels with a tendency to syneresis due to a change in macromolecule conformation from a random coil to a double helix when cooling hot aqueous solutions. The introduction of Ca^2+^ ions into its composition is able to increase the strength of gels due to the increase in the small soluble cluster-associated “domains” by the interaction of spirals from different “domains” with the participation of cations with the formation of bridges between adjacent double chains through electrostatic bonds between sulfate groups. The introduction of galactomannan solution into the carrageenan leads to the formation of a synergistic gel system due to hydrogen bonds. This synergism is demonstrated on the basis of experimentally obtained data, according to which the variation in GM-AgNPs/CG-AgNPs ratios is accompanied by changes in the elastic modulus of hydrogels and strain–stress dynamics.

### 2.6. Investigation of AgNPs Release Dynamics from Hydrogels Based on the GM-CG System

**Figure 8:**
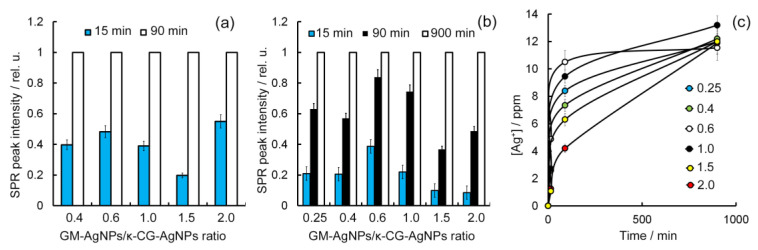
AgNPs release dynamics from hydrogels based on the GM-AgNPs/κ-CG-AgNPs system with different composite ratios in the absence (**a**) and presence of Ca^2+^ ions (**b**); Ag+ concentration dynamics in aqueous medium within the dissolution of hydrogels based on the GM-AgNPs/κ-CG-AgNPs system in the presence of Ca^2+^ ions with different composite ratios (**c**). Error bars are hidden in the bar when not visible; data are mean ± SD, *n* ≥ 3.

Despite the frequent evidence of the successful use of natural polysaccharides, including GM and κ-CG, as transdermal drug delivery agents, the question of the possibility of AgNP release from hydrogel is genuine [64,65,66]. This is primarily due to the fact that the antiradical activity of the nanomaterials obtained in this work is conditioned exclusively by AgNPs. The role of polysaccharide in the already obtained composite materials is to provide their aggregative stability and nanodispersity, as well as the realization of gel-forming and water-holding properties that underlie the possibility of creating AgNP-containing hydrogels on their basis. The question of possible transdermal transfer of AgNPs from the hydrogel matrix requires further detailed study, including the use of laboratory animals. The study of AgNP release from hydrogel matrix based on the GM-AgNPs-κ-CG-AgNPs system can be easily carried out using the SPR phenomenon as a parameter to evaluate the degree of AgNP release. Above are the absorption spectra of aqueous solutions of GM-AgNPs and κ-CG-AgNPs composites, which demonstrate the presence of an intense plasmonic absorption maximum in the visible region of the spectrum, which is retained in the hydrogels, obtained on their basis. This study was also carried out at 30 °C for the time required for the complete dissolution of the hydrogel in water and transfer of all nanoparticles into solution. It was found that in the case of testing the hydrogel GM-AgNPs/κ-CG-AgNPs with a 1:1 ratio of components (which also showed the best water-holding capacity), the most pronounced (55%) release of AgNPs was observed already within 15 min after placing the sample in water. The lowest (20%) release of AgNPs was observed in the case of the composite sample GM-AgNPs/κ-CG-AgNPs at a ratio of 1.5 (Figure 8a). After 1.5 h, the complete dissolution of hydrogels was observed irrespective of the ratio of components with the complete 100% release of AgNPs into the solution. Further increase in the observation duration was not accompanied by an increase in the SPR band’s intensity.

The introduction of Ca^2+^ cations into the hydrogels was accompanied by a significant change in the nanoparticles’ release dynamics into the solution. Fifteen minutes after the introduction of GM-AgNPs-κ-CG-AgNPs—Ca^2+^ hydrogels into the aqueous solution, there was observed almost the complete absence of gel dissolution signs and the appearance of only weak yellow-brown staining of the solution due to the transition of a small amount of AgNPs into the solution. The maximum amount (34%) of released AgNPs was recorded in the case of testing hydrogels with the ratio of GM-AgNPs/κ-CG-AgNPs 0.6 (Figure 8), whereas the lowest release (8.6%) was recorded for the system GM-AgNPs/κ-CG-AgNPs in the ratio of 2. After 1.5 h, in contrast to hydrogels obtained without the addition of Ca^2+^ cations, the complete dissolution of hydrogels was also not observed. The extraction solutions were characterized by an increase in yellow-brown staining intensity. The most complete (83%) release of AgNPs was also observed for the system GM-AgNPs/κ-CG-AgNPs in the ratio 0.6, and the least complete (36%) for GM-AgNPs/κ-CG-AgNPs hydrogels in the ratio of 2. Complete dissolution of hydrogels irrespective of the ratio of components was observed after 15 h of exposure. The significantly lower solubility of hydrogels and the prolonged release of AgNPs into solution was probably due to the strong binding of κ-CG double helices in the process of structuring and gel formation, as well as the impossibility of free escape of GM-AgNPs from internal cavities in the three-dimensional gel structure based on κ-CG. Presumably, such a stepwise release of AgNPs from hydrogels into the aqueous medium may be due to the different involvement of GM and κ-CG macromolecules in the formation of the three-dimensional hydrogel structure. Considering the pronounced gel-forming ability of CG, especially intensified in the presence of Ca^2+^ ions in this case, the mobility of its macromolecules is significantly more limited compared to that of the GM macromolecules. Therefore, in the case of GM-AgNPs/κ-CG-AgNPs hydrogels with a component ratio of 0.4 (or 0.25–0.4 in the presence of Ca^2+^ cations), the initial release of AgNPs into the solution may be due to the release of the GM-AgNPs composite with the subsequent destruction of the spatial structure of the three-dimensional gel network and dissolution of κ-CG-AgNPs.

The most intensive release of AgNPs was observed for hydrogels with a GM-AgNPs/CG-AgNPs ratio of 0.6 regardless of the presence of Ca^2+^, whereas in the case of calcium cations addition, a significant inhibition of the silver release rate was observed even in cases of high (1.5–2.0) GM-AgNPs/CG-AgNPs ratios and even after 1.5 h of incubation. This may be due to a change in the mechanism of formation of the three-dimensional spatial gel structure itself, including the possible formation of an interpenetrating three-dimensional network of both GM and κ-CG macromolecules.

Spectrophotometric data were confirmed by ICP-MS data on the detection of silver concentration change (after preliminary mineralization of samples) in solutions containing GM-AgNPs/CG-AgNPs/Ca^2+^ hydrogels within similar time intervals (Figure 8c). It is shown that the smoothest release of silver is characteristic for hydrogels with the ratio GM-AgNPs/CG-AgNPs 2.0. This ratio of components provides not only a pronounced moisture-holding ability of hydrogels, but also a smooth and continuous release of silver to maintain its optimal concentration on the treated surface. In addition, the high content of GM-AgNPs in the hydrogel composition is able to provide more pronounced antiradical properties of the obtained hydrogels due to the high antiradical activity of GM-AgNPs composites themselves.

The obtained results indicate and confirm the previously established possibilities of AgNPs (or drug substance) release from the polysaccharide hydrogel matrix. The dynamics of release in this case is determined to a greater extent by the presence of Ca^2+^ ions providing the formation of a stronger intermolecular framework of aggregated double helices κ-CG preventing the rapid release of AgNPs from the hydrogel and the transition of the hydrogel itself into the solution. According to the literature data, macromolecules including natural polysaccharides and GM and κ-CG are able to penetrate skin barriers, facilitating the penetration of drug substances into the body. However, the realization in this case of such a mechanism of action of hydrogels obtained in the framework of this work requires further intensive studies and will be the subject of further research.

## 3. Conclusions

Thus, using the methods of “soft chemistry”, a number of water-soluble AgNPs based on polysaccharide macromolecules GM and κ-CG acting simultaneously as a silver-reducing agent to the zero-valent state and a stabilizer of the formed nanoparticles were obtained. The prevailing preservation of the polymeric structure of polysaccharides during the synthesis process determines the presence of the whole range of rheological (water-solubility, gel-forming, and water-holding capacity) properties that are characteristic for them and for GM-AgNPs and κ-CG-AgNPs composites synthesized on their basis. Using spectrophotometric techniques and a standard cation radical as a model, the radical-binding capacity of all the obtained composites was investigated. It was found that GM-AgNPs composites are characterized by more pronounced antiradical activity against ABTS compared to κ-CG-based composites containing 2.5% Ag. Varying the ratio of 1.5% aqueous solutions of GM-AgNPs/κ-CG-AgNPs composites from 0 to 4 and introducing Ca^2+^ cations allowed us to obtain a series of GM-AgNPs-κ-CG-AgNPs hydrogel systems with varying values of the water-holding capacity, elastic modulus, and dynamics of silver release from the hydrogel. It was found that an increase in water-holding capacity was observed for hydrogels with GM-AgNPs/κ-CG-AgNPs ratios of 1 or 0.25 when Ca^2+^ cations were added. The prolonged and complete release of AgNPs from hydrogels was provided when Ca^2+^ cations were added and when the composite ratio of GM-AgNPs/κ-CG-AgNPs was 1.5–2.0, while hydrogels with the highest value of elastic modulus were formed at a 0.6–1.5 ratio of these composites. Taking into account the pronounced antiradical activity of GM-AgNPs and κ-CG-AgNPs composites and the high water-holding capacity of GM-AgNPs-κ-CG-AgNPs hydrogels obtained on their basis, in combination with the step-by-step prolonged release of AgNPs from their composition, the development of dressing materials with combined antimicrobial and antiradical actions on their basis seems to be especially promising.

## 4. Materials and Methods

### 4.1. Materials and Reagents

In this work, we used galactomannan locust bean gum mark (Mw 2300 kDa, CP Kelco, Lille Skensved, Denmark) and κ-carrageenan WR-78 mark (Mw 1800 kDa, CP Kelco, Lille Skensved, Denmark), which were previously subjected to partial alkaline depolymerization according to the procedure described in detail in [67]. The resulting GM and κ-CG samples exhibited improved water solubility and reduced Mw values to 1300 kDa and 1100 kDa, respectively. GM: Found, %: C 46.64; H 6.37; O 46.99. IR spectra (KBr, ν, cm^−1^): 3430 (OH), 2927 (C-H), 1210–1020 (C-C, C-O), 872, and 811 (β-glycoside bonds of mannopyranose units). κ-CG: Found, %: C 32.04; H 6.10; O 52.44; S 6.08; K 3.34; Na 3.67. IR spectra (KBr, ν, cm^−1^): 3560, 3422 (OH), 2970, 2942, 2913 (C-H), 1200-1000 (C-O), 910, 771 (β-glycosidic bond of pyranose rings), and 850 (SO_3_). All commercial reagents AgNO_3_, NaOH, ethyl alcohol (96%) (all Vecton (Russia, Saint Petersburg)), and ABTS^+·^ (2,2′-azino-bis (3 ethylbenzothiazoline-6-sulphonic acid) (Sigma-Aldrich, Burlington, MA, USA) were used without additional purification.

### 4.2. Characterizations

The X-ray diffraction study was carried out on a Bruker D8 ADVANCE (Ettlingen, Germany) X-ray diffractometer with Cu Kα—radiation mode Locked Coupled. The exposure time was 1 s for the phase analysis and 3 s for the cell parameters and coherent lengths.

Microphotographs were obtained on a Leo 906 E transmission electron microscope using copper grids with a formvar layer as a substrate. The particle size measurement was performed manually using the IPWin45 program (Version 4.5.0.29).

The elemental composition of the nanocomposites was obtained on a Hitachi TM 3000 electron scanning microscope (Angstrom Scientific Inc., Ramsey, NJ, USA) with an SDD XFlash 430-4 X-ray detector using X-ray energy dispersive microanalysis and on a Flash 2000 CHNS analyzer (Thermo Fisher Scientific, Waltham, MA, USA).

Hydrodynamic radii (Rh) were determined using the method of dynamic light scattering (DLS) on a Photocor Compact-Z correlation spectrometer (Photocor, Moscow, Russia) (light source–thermostabilized semiconductor laser of 20 mW power with wavelength λ = 638 nm) at an angle of 90°. The correlation function was analyzed using the Dynals (Version 2.0) program for data processing of dynamic light scattering. Solutions for analysis were prepared by dissolving, at room temperature for eight hours, 5 mg of sample in 10 mL of distilled water, pre-filtered through a syringe filter for dedusting. The time for each measurement was at least 200 s. The measurement was performed in triplicate.

IR spectra were recorded on a Bruker Vertex 70 FTIR spectrometer (RAM II) (Bruker, Ettlingen, Germany) in KBr tablets in the range of 4000–400 cm^−1^.

Optical absorption spectra of 0.03% aqueous solutions of nanocomposites were recorded on a Perkin Elmer Lambda 35 spectrophotometer (PerkinElmer, Waltham, MA, USA) with respect to distilled water in a 1 cm quartz cuvette in the wavelength range of 190–1000 nm.

All rheological measurements were performed in triplicate for each sample according to the methodology [39] using a HAAKE Viscotester iQ rheometer (Thermo Scientific) in a cell with parallel plate geometry. Hydrogel samples, 1.3 cm in diameter and 1.3 cm in height, pre-thermostated at the measurement temperature (room temperature) for 4 h, were used for the study.

### 4.3. Procedure for Synthesis of GM-AgNPs and κ-CG-AgNPs Nanocomposites

Nanocomposites were synthesized according to the protocol described in detail in [45], with minor modifications. Briefly: to a solution of 1 g of GM or κ-CG in 120 mL of H_2_O dist. under stirring was added 10 mL of aqueous solution containing 40–331 mg of AgNO_3_. The resulting mixture was incubated at room temperature for 10 min, after which the pH of the reaction medium was adjusted to 10.2–10.8 by adding 1N aqueous NaOH solution followed by incubation in a water bath (70 °C) for 20–50 min (depending on the percentage of silver in the composite). The isolation of the target products and purification of impurities was carried out by precipitation of the reaction medium in a 4-fold excess of ethyl alcohol, followed by repeated washing and separation of the precipitate on a Schott filter and air-drying at room temperature. The yield was 82–93%. The silver conversion from Ag^+^ to Ag^0^ was near to 100% (98–100%) in all cases. More detailed synthesis conditions for each nanocomposite sample are presented in Table 2.

GM-AgNPs (2.5%): Found, %: C 39.34; H 6.29; O 51.87; Ag 2.5. IR spectra (KBr, ν, cm^−1^): 3432 (OH), 2931 (C-H), 1204–1019 (C-C, C-O), 871, and 812 (β-glycoside bonds of mannopyranose units). GM-AgNPs (4.0%): Found, %: C 38.62; H 6.01; O 51.37; Ag 4.0. IR spectra (KBr, ν, cm^−1^): 3434 (OH), 2925 (C-H), 1210–1020 (C-C, C-O), 870, and 809 (β-glycoside bonds of mannopyranose units). GM-AgNPs (7.0%): Found, %: C 35.81; H 5.88; O 51.31; Ag 7.0. IR spectra (KBr, ν, cm^−1^): 3430 (OH), 2920 (C-H), 1210–1020 (C-C, C-O), 877, and 812 (β-glycoside bonds of mannopyranose units). GM-AgNPs (8.6%): Found, %: C 37.78; H 5.35; O 48.27; Ag 8.6. IR spectra (KBr, ν, cm^−1^): 3431 (OH), 2932 (C-H), 1212–1018 (C-C, C-O), 877, and 810 (β-glycoside bonds of mannopyranose units). GM-AgNPs (12.0%): Found, %: C 33.21; H 4.93; O 49.86; Ag 12.0. IR spectra (KBr, ν, cm^−1^): 3429 (OH), 2921 (C-H), 1210–1020 (C-C, C-O), 872, and 810 (β-glycoside bonds of mannopyranose units). GM--AgNPs (17.0%): Found, %: C 29.72; H 4.31; O 48.97; Ag 17.0. IR spectra (KBr, ν, cm^−1^): 3430 (OH), 2920 (C-H), 1210-1020 (C-C, C-O), 871, and 811 (β-glycoside bonds of mannopyranose units).

κ-CG-AgNPs (2.5%): Found, %: C 28.77; H 5.01; O 51.57; Ag 2.5; Na 3.04; K 3.1; S 6.01. IR spectra (KBr, ν, cm^−1^): 3564, 3420 (OH), 2975, 2940, 2911 (C-H), 1200–1000 (C-O), 911, 770 (β-glycosidic bond of pyranose rings), 850 (SO_3_); κ-CG-AgNPs (4.8%): Found, %: C 26.06; H 4.98; O 51.69; Ag 4.8; Na 3.14; K 3.21; S 6.12. IR spectra (KBr, ν, cm^−1^): 3560, 3420 (OH), 2970, 2942, 2910 (C-H), 1200–1000 (C-O), 912, 770 (β-glycosidic bond of pyranose rings), 850 (SO_3_); κ-CG-AgNPs (7.0%): Found, %: C 26.04; H 5.00; O 49.75; Ag 7.0; Na 3.12; K 3.05; S 6.04. IR spectra (KBr, ν, cm^−1^): 3561, 3419 (OH), 2972, 2940, 2911 (C-H), 1200–1000 (C-O), 910, 775 (β-glycosidic bond of pyranose rings), 850 (SO_3_); κ-CG-AgNPs (8.6%): Found, %: C 25.51; H 4.92; O 48.54; Ag 8.6; Na 3.10; K 3.03; S 6.3. IR spectra (KBr, ν, cm^−1^): 3560, 3422 (OH), 2971, 2944, 2910 (C-H), 1200–1000 (C-O), 911, 770 (β-glycosidic bond of pyranose rings), 850 (SO_3_); κ-CG-AgNPs (12.0%): Found, %: C 24.02; H 4.94; O 46.80; Ag 12.0; Na 3.04; K 3.1; S 6.1. IR spectra (KBr, ν, cm^−1^): 3563, 3421 (OH), 2970, 2940, 2914 (C-H), 1200–1000 (C-O), 912, 772 (β-glycosidic bond of pyranose rings), 850 (SO_3_).

### 4.4. ABTS Radical-Scavenging Activity

The antiradical activity of GM-AgNPs and κ-CG-AgNPs composites against the ABTS cation-radical was measured as described by Lesnichaya et al. [68]. The ABTS^+·^ cation-radical inhibition (%) was calculated by the following Equation (1):

(1) $$ABTS\;scavenging\;ability\;\left(\%\right)=\left(1-\left(\frac{A_{a}-A_{b})}{A_{c}}\right)\right)\times 100\%$$

where *A_a_* is the absorbance of the sample mixed with the ABTS^+·^ solution, *A_b_* is the absorbance of the sample without the ABTS^+·^ solution, and *A_c_* is the absorbance of the ABTS^+·^ solution without the sample as a blank control.

The experiment was carried out in triplicate. The results were averaged.

### 4.5. Preparation of GM-AgNPs-κ-CG-AgNPs Hydrogels

GM-AgNPs and κ-CG-AgNPs composites containing 2.5% AgNPs were used to obtain hydrogels. According to XRD and TEM, these composites are characterized by the smallest size of formed nanoparticles. Hydrogels were obtained by mixing their 1.5% aqueous solutions in different GM/κ-CG ratios varying from 0 (only κ-CG-AgNPs composite) to 0.25, 0.4, 0.6, 1.0, 1.5, 2, and 4 in order to obtain samples with satisfactory moisture retention capacity and to modify the strength and elasticity of hydrogels. In addition, a series of hydrogels with a similar ratio of components but with the addition of calcium chloride in the amount of 0.0028 mol per 1g of nanocomposite was also obtained. The hydrogels were obtained using the preliminary preparation of 1.5% solutions of composites at 35° C for 3 h with the subsequent mixing of composites and introduction of calcium chloride solution in the proportions noted above. After thorough mixing, the tubes with the mixture were placed in a thermostat (temperature 7 °C) for gel formation. Gel formation was evaluated after 4 h. 

### 4.6. Study of the Water-Holding Capacity of Hydrogels Based on the GM-AgNPs-κ-CG-AgNPs System

The water-holding capacity of hydrogels was estimated gravimetrically by fixing the hydrogel mass loss during its storage in the thermostat at atmospheric pressure, moisture, and temperature at 30 °C. Measurements were carried out daily for 12 days at the same time. The wet weight of the hydrogels was recorded at each time interval. The weight loss was calculated using Equation (2).

(2) $$∆m,\;\%=\left(\frac{m_{0}-m_{i}}{m_{0}}\right)\times 100$$

where ∆*m* is the mass loss, *m*_0_ is the initial hydrogel mass, and *m_i_*is the hydrogel mass on the day of measurement. The study was performed in three repeats.

### 4.7. Study of AgNPs Release Dynamics from Hydrogels in Aqueous Medium

The dynamics of AgNP release from hydrogels of different compositions was investigated spectrophotometrically by detecting the intensity of the plasmon absorption band in an aqueous solution containing the sample under study. An amount of 0.1 g of freshly prepared hydrogel was placed in a test tube containing 15 mL of distilled water, and the absorbance of the solutions in the visible region of the spectrum was measured after 15 min, 1.5 h, and 15 h of placing the hydrogel in water.

In addition, the dynamics of silver release from Ca^2+^-added hydrogels (which showed the most promising properties) was investigated using inductively coupled plasma mass spectrometry (ICP-MS) after preliminary mineralization of samples in a mixture of concentrated nitric acid and H_2_O_2_. Sample preparation was carried out using the protocol described in detail in Ref. [69]. The analysis was performed on an Aurora M90 mass spectrometer (Bruker Scientific Instruments, Billerica, MA, USA).

## Figures and Tables

**Table 1:** Main characteristics of the crystalline phase of silver-containing nanocomposites based on GM and κ-CG.

Object	Ag, %	Lattice Parameter a, nm	The Average Size of the Crystallites, nm	D*_av_* TEM	S*_sp_* cm^2^/cm^3^	Surface Atoms Content, %
GM-Ag	2.5	0.4077 (2)	5.1		1.2 × 10^7^	30
GM-Ag	4.0	0.4075 (3)	6.5	6.7	9.2 × 10^6^	24
GM-Ag	7.0	0.4081 (5)	7.2	7.3	8.3 × 10^6^	23
GM-Ag	8.6	0.4086 (6)	8.6		7.0 × 10^6^	19
GM-Ag	12	0.4088 (2)	11.4		5.2 × 10^6^	14
GM-Ag	17.0	0.4086 (4)	18.3	21.0	3.3 × 10^6^	9
κ-CG-Ag	2.5	0.4068 (2)	6.4		9.4 × 10^6^	25
κ-CG-Ag	4.8	0.4065 (3)	8.3	10.7	7.2 × 10^6^	20
κ-CG-Ag	7.0	0.4077 (5)	15.1	12.2	4.0 × 10^6^	11
κ-CG-Ag	8.6	0.4088 (5)	21.0		2.8 × 10^6^	8
κ-CG-Ag	12.0	0.4078 (2)	24.8	24.8	2.4 × 10^6^	7

**Table 2:** Methodological details of the synthesis of polysaccharide-stabilized AgNPs based on GM and κ-CG matrices.

Code	% Ag	Ag^+^/Polysaccharide Ratio	Synthesis Time, min
GM-AgNPs	2.5%	0.025 (40 mg AgNO_3_ per 1 g polysaccharide)	20
GM-AgNPs	4.0%	0.041 (65 mg AgNO_3_ per 1 g polysaccharide)	25
GM-AgNPs	7.0%	0.075 (118 mg AgNO_3_ per 1 g polysaccharide)	30
GM-AgNPs	8.7%	0.095 (150 mg AgNO_3_ per 1 g polysaccharide)	35
GM-AgNPs	12.0%	0.14 (220 mg AgNO_3_ per 1 g polysaccharide)	40
GM-AgNPs	17.3%	0.21 (331 mg AgNO_3_ per 1 g polysaccharide)	50
κ-CG-AgNPs	2.5%	0.025 (40 mg AgNO_3_ per 1 g polysaccharide)	20
κ-CG-AgNPs	4.8%	0.05 (79 mg AgNO_3_ per 1 g polysaccharide)	25
κ-CG-AgNPs	7.0%	0.075 (118 mg AgNO_3_ per 1 g polysaccharide)	30
κ-CG-AgNPs	8.6%	0.095 (150 mg AgNO_3_ per 1 g polysaccharide)	35
κ-CG-AgNPs	12.0%	0.14 (220 mg AgNO_3_ per 1 g polysaccharide)	40

## Data Availability

The original contributions presented in this study are included in the article. Further inquiries can be directed to the corresponding author.
